# Gold-Nanoparticle-Based Chiral Plasmonic Nanostructures and Their Biomedical Applications

**DOI:** 10.3390/bios12110957

**Published:** 2022-11-01

**Authors:** Hanbo Li, Xinshuang Gao, Chenqi Zhang, Yinglu Ji, Zhijian Hu, Xiaochun Wu

**Affiliations:** 1 CAS Key Laboratory of Standardization and Measurement for Nanotechnology, National Center for Nanoscience and Technology, Beijing 100190, China; 2 School of Nanoscience and Technology, University of the Chinese Academy of Sciences, Beijing 100049, China

**Keywords:** chirality, surface plasmon, gold nanoparticle, plasmonic circular dichroism

## Abstract

As chiral antennas, plasmonic nanoparticles (NPs) can enhance chiral responses of chiral materials by forming hybrid structures and improving their own chirality preference as well. Chirality-dependent properties of plasmonic NPs broaden application potentials of chiral nanostructures in the biomedical field. Herein, we review the wet-chemical synthesis and self-assembly fabrication of gold-NP-based chiral nanostructures. Discrete chiral NPs are mainly obtained via the seed-mediated growth of achiral gold NPs under the guide of chiral molecules during growth. Irradiation with chiral light during growth is demonstrated to be a promising method for chirality control. Chiral assemblies are fabricated via the bottom-up assembly of achiral gold NPs using chiral linkers or guided by chiral templates, which exhibit large chiroplasmonic activities. In describing recent advances, emphasis is placed on the design and synthesis of chiral nanostructures with the tuning and amplification of plasmonic circular dichroism responses. In addition, the review discusses the most recent or even emerging trends in biomedical fields from biosensing and imaging to disease diagnosis and therapy.

## 1. Introduction

Chirality reflects the asymmetry of an object, which cannot coincide with its mirror image via translation and rotation. It widely exists in nature from small molecules to its presence in the vast universe. For example, the helical structure of DNA molecules, the vine of morning glory, the shell of snails, and the spiral nebula all exhibit chirality [1]. The two mirror images of opposite chirality are called left- and right-handedness. Enantiomers interact differently with left circularly polarized light (LCP) and right circularly polarized light (RCP), which causes the different absorption of RCP and LCP light and leads to circular dichroism (CD), often used for the characterization of molecule chirality [2]. The optical activity was first discovered by Arago in quartz and biot in certain liquids or gases in 1811 [3]. Before 1998, most chiral materials synthesized and studied have been based on organic molecules, including chiral-organic-molecule-based systems displaying microscale-length chiral structures [4]. Chirality at the molecular level, for instance, protein, sugar, and synthetic molecules, is well understood, but it has yet been elucidated upon when extending to nanoscales and above [5,6]. Recently, chirality research expanded to nanoscience fields, especially to nanoplasmonics, which focuses on new phenomena/effects produced by surface plasmon resonance (SPR). At nanoscales, strong chiral interactions between light and plasmonic materials not only brings novel plasmonic chiral effects but also generates new applications. Plasmonic chirality is often characterized using plasmonic circular dichroism (PCD), which is related to circular dichroism responses at a localized SPR (LSPR) of plasmonic nanostructures [7]. As a kind of important plasmonic nanomaterials, metal nanomaterials have advantages of strong and tunable LSPR. The peak position, intensity, and absorbance/scattering ratio are closely related to composition, shape, size, structure, and local dielectric environment, thus posing a huge tuning space. In addition, the local electromagnetic field upon plasmon excitation brings in enhanced matter–light interactions at the nanoscale. The high tunability of the LSPR and its strong local electromagnetic field endow plasmonic chiralities with wide spectral responses and strong optical activities, promising potential applications in drug delivery, medical diagnosis, and biosensors [8]. Thanks to the rapid advancement of wet chemical syntheses of metal nanoparticles (NPs), recent progress shows large improvements in chiroptical properties and even gives birth to a new branch of chemical nanoplasmonics. Despite the fact that mechanisms and the precise maneuverer of cross-length chirality transfers and amplification are in exploratory stages [9,10], chiral plasmonic brings new vitality to the traditional chirality field.

Chiral plasmonic mainly focuses on the fabrication of chiral plasmonic nanomaterials, the modulation of their plasmonic chirality, and the development of potential applications. Chiral plasmonic nanomaterials can be categorized into two types: discrete chiral NPs/nanostructures and chiral superstructures and assembly structures of achiral/chiral plasmonic NPs themselves or their hybrid structures with other NPs. The former can be obtained either via attaching chiral molecules on the surface of achiral plasmonic NPs or via the chiral molecule-guided chiral growth of NPs with obvious chiral shapes [11]. Due to weak molecule dipole–plasmon dipole interactions, the simple chiral molecule-attaching method often results in weak plasmonic optical activity [12,13]. In order to enhance this interaction, the design idea of hotspots is introduced by encapsulating chiral molecules in the core–shell interface or nano-cavity of plasmonic nanostructures via overgrowth strategy [14]. In contrast, the latter with the formation of chiral shapes can produce substantial chiroptical responses. Recently, wet chemical strategies have made great progress in the synthesis of such discrete plasmonic NPs with well-controlled chiral morphologies [15]. Chiral plasmonic superstructures are mainly constructed using the bottom-up assembly of achiral plasmonic NPs under the guide of various chiral molecules/templates and have been intensively investigated [16]. Small chiral molecules often act as chiral linkers to connect NPs or as chirality-driven molecules upon adsorption on particle surfaces, such as amino acids [17,18,19,20,21,22,23], chiral polymer molecules [24], DNA/RNA [25,26,27], peptides, and small protein molecules [28,29,30,31]. Due to the existence of hotspots in assemblies, the contribution of chiral molecule dipole and plasmon dipole may be involved [32]. Chiral templates/media on the other hand guide the arrangement of plasmonic NPs in chiral configurations, such as long chiral fibers [33,34], liquid crystals [35,36,37,38], hydrogels [39,40], protein aggregates [40,41], and DNA origami [42,43,44] to name a few. In particular, the development of DNA origami has made it possible to precisely control the spatial arrangement and structural modulation of plasmonic NPs and, thus, led to chiral plasmonic nanostructures with strong chiroptical signals. Due to the high dependence of optical activities on assembly structures, a small structural change may lead to large changes in chiral signals and, thus, provide the basis for the development of biosensing platforms based on chiral plasmonic nano-assemblies. Chiral plasmonic nanomaterials demonstrated great potentials ranging from bio-sensing and imaging to disease diagnoses and treatments [45,46,47].

In this review, using a typical plasmonic nanomaterial of gold NPs, we summarize recent progressions in chiral plasmonic nanomaterials and their biomedical applications. It includes five parts. A short history of plasmonic chirality is given in the introductory section, followed by the fabrication of two major types of chiral plasmonic nanomaterials via wet chemical methods in the second section. Then, the unique plasmonic features in combination with chirality are emphasized in the third section. Biomedical applications including bio-detection, bio-imaging, disease diagnoses, and treatment are discussed in the fourth section. In the end, a brief summary and future perspective are given in the fifth section.

## 2. Wet-Chemical Fabrication of Chiral Plasmonic Nanomaterials

As mentioned in the Introduction, discrete chiral plasmonic nanostructures and chiral plasmonic superstructures are two major types of chiral plasmonic nanomaterials, which are generated mainly using wet chemical synthesis and self-assembly process, respectively.

### 2.1. Discrete Chiral Plasmonic NPs/Nanostructures

Seed-mediated growth plays an important role in the size and shape tuning of plasmonic NPs. This is not an exception with respect to the synthesis of chiral NPs [48,49,50,51,52,53,54]. Based on seed-mediated growth, there are often two representative routes for obtaining chiral NPs [11]. Using achiral NPs as seeds, chiral molecules first adsorb onto the seed’s surface and later are encapsulated in the seed–shell interface or shell cavities during the shell’s growth. The obtained core–shell nanostructures show obvious chiroptical activities but do not necessarily expose chiral morphologies for the shell. The hotspot-enhanced chiral interaction may be the main source of PCD responses. Similarly, also using achiral NPs as seeds, chiral-molecule-mediated shell growth can lead to the formation of three-dimensional (3D) chiral morphology.

#### 2.1.1. Strong Chiral Ligand-Mediated Synthesis Strategy

**Figure 1:**
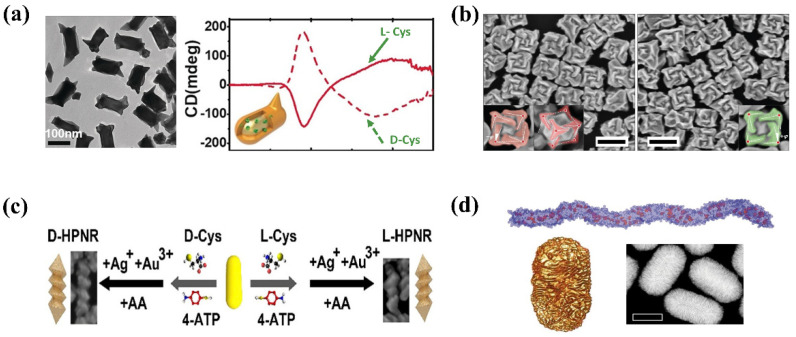
Synthesis of discrete chiral plasmonic NPs/nanostructures. (**a**) TEM image and CD spectra of c-Au NRs [56]. Scale bars, 100 nm. Copyright 2018, Wiley. (**b**) SEM images of L-Cys- (left) and D-Cys-mediated gold NPs (right) [15]. ±φ is the tilt angle. Scale bars, 200 nm. Copyright 2018, Springer Nature. (**c**) Schematic of HPNR growth process [57]. Copyright 2021, American Chemical Society. (**d**) MD simulation of R-BINOL-CTAC structure, TEM tomography, and SEM images of grooved chiral Au NRs [58]. Scale bars, 100 nm. Copyright 2020, American Association for the Advancement of Science.

As a simple and easy strategy, different chiral NPs with a metal-chiral molecule–metal structure have been demonstrated. For instance, using gold nanorods (AuNRs) as seeds, our group obtained starfruit-like Au NPs with the help of chiral cysteine (Cys)-modulated Au overgrowth [55]. The optimized anisotropic g factor can reach −0.005. Furthermore, the unique role of Cys pre-incubation was revealed. Pre-adsorbed Cys molecules mainly affected shell overgrowth mode. In contrast, Cys molecules located at the hotspots of the Au shell contribute to strong PCD responses. Moreover, when seeds changed from AuNRs to Au nanospheres, no PCD response was observed, signifying the effect of core anisotropy on chiroptical activity. Using a similar procedure, in the presence of L-Cys and D-Cys, Zheng et al. obtained AuNRs with chiroptical responses (c-AuNRs) (Figure 1a) [56]. By tuning Cys concentrations, c-Au NRs with different morphologies were achieved, such as a conformal shell at low Cys concentration (4 nM), a shell with one or two spikes at rod ends at medium Cys concentration (40 nM), or a shell with multiple spikes protruding from the rod seed at high Cys concentration (400 nM). Among them, the c-Au NRs with one or two spikes exhibited large PCD responses, attributed to the proper encapsulation of Cys molecules at hotspot cavities between the core and the shell (Figure 1a Inset). The morphology’s evolution was mainly determined by the surface blocking of adsorbed thiols. Apart from enhanced dipole–dipole interactions, the formation of local chiral surface morphologies was not discussed.

The breakthrough in the formation of 3D chiral NPs occurred in 2018. During this year, Nam et al. reported the successful synthesis of 3D gold helicoids with giant PCD responses for the first time [15]. Very interestingly, it was a seed-mediated growth strategy. It is not so much an accident as a spur with long-term accumulation. Using achiral cubic or octahedral nanogolds as seeds, under the guide of chiral ligands, such as cysteine or cysteine-based peptides, one hundred nanometer-sized chiral gold nanoparticles were obtained with adjustable chiral plasmonic resonance (Figure 1b). Under optimized growth conditions, chiral NPs with a dissymmetry factor of ~0.2 were prepared. The formation of high Miller index surfaces ({hkl}, h ≠ k ≠ l ≠ 0) was regarded as the source of chirality. The enantioselective interactions between chiral molecules and surfaces of NPs resulted in different growth rates of chiral facets and eventually the formation of helicoid NPs. When extending this strategy to synthesize chiral palladium (Pd) NPs, Nam and co-workers found that the obtained Pd NPs had no evident PCD responses [48]. SEM images observed the formation of spiral Pd structures, either clockwise or counterclockwise rotation, on gold cubic seeds. However, the chiral bias for Pd NPs was much lower than that for Au NPs, possibly due to weaker interactions between thiol and Pd surfaces and the low chiral specificity of chiral thiols to high-index Pd surfaces. Following the pioneering work of Nam’s group, more discrete 3D chiral plasmonic nanostructures have been reported [50,52,54]. For instance, our group developed an achiral thiol-assisted growth strategy to synthesize discrete helical plasmonic nanorods (HPNRs) with strong and tunable PCD responses [57]. AuNRs, after pre-incubated with the mixture of Cys and 4-aminothiophenol (4-ATP), were used as seeds to support the overgrowth of helical Au and Ag alloy shells (Figure 1c). Via the optimization growth parameters, large PCD responses with a g factor value of ~0.04 were achieved. Cys was suggested to play a crucial role in the formation of the helical morphology, while 4-ATP mainly reduced the structural defects and thus improved PCD signals. Finite-difference time-domain (FDTD) simulations verified that PCD responses come from the helical structure, and this is in line with experimental results. The combination of chiral and achiral thiols enriched the synthesis choice of chiral NPs. Using gold nanooctopods (NOPs) as seeds and in the presence of GSH, Liu et al. demonstrated an eight-step overgrowth for the formation of chiral gold NOPs, which have a propeller-like structure with eight arms bending from 〈111〉 to 〈100〉 directions [54].

#### 2.1.2. Weak Chiral Ligand-Mediated Synthesis Strategy

Inspired by chiral micelles, Liz-Marzan et al. developed another synthesis strategy to grow chiral NPs [58]. Chiral cosurfactants, such as R-/S-1.1′-bi(2-naphthol) (BINOL) and R-/S-1.1′-binaphthyl-2.2′-diamine (BINAMINE), were used to induce worm-like chiral micelles, similarly via a “sergeants and soldiers” principle often used in forming supramolecular chiral assemblies (Figure 1d). By introducing them to the growth solution containing the micelle of cetyltrimethylammoniumchloride (CTAC), they obtained grooved chiral Au NPs via conventional seed-mediated growth. They speculated that the adsorption of chiral micelles on AuNR surface forms chiral and worm-like aggregates, as confirmed by molecular dynamics (MD) simulations. Chiral structures of the aggregates are transferred to the deposited metal shells during the growth process. Furthermore, the dimensions of Au NRs can be used to tune plasmonic CD responses. The maximum g factor of ~0.2 was comparable to those obtained from chiral thiol-mediated NPs [15]. When changing gold ions to Pt ions, the Pt shell containing chiral wrinkles was also obtained. The chiral co-surfactant- templated growth obviously provides another useful route for the synthesis of discrete chiral NPs.

#### 2.1.3. Other Synthesis Strategies

Quite recently, by introducing circularly polarized light (CPL) to the synthesis of chiral plasmonic NPs, Xu et al. further pushed the achieved chiroptical activity to a new level (g factor: ~0.44) for chemically synthesized discrete chiral plasmonic nanostructures [59]. Using Au nanoprisms as seeds and in the presence of chiral cysteine-phenylalanine (CYP) dipeptides, chiral Au NPs were grown under the irradiation of light at wavelengths overlapping with the plasmonic resonance of seeds. Using L-CYP, L-P^+^ NPs assisted with LCP light showed the strongest CD signal, following with L-P^0^ NPs under linearly polarized light and L-P^−^ NPs with RCP light, respectively. L- NPs without light irradiation exhibited the weakest CD signal. Using D-CYP, the opposite CD sign was observed with a g-factor magnitude of D-P^−^ NPs > D-P^0^ NPs > D-P^+^ NPs > D-NPs. The results indicated that the light-assisted chiral particle growth is accompanied with the chirality transfer from photons to NPs. L-P^+^ NPs and D-P^−^ NPs showed propeller structures with blades exhibiting anticlockwise and clockwise rotations, respectively. Although the handedness of NPs was controlled by chiral ligands, the curvature depth of blades was determined by illumination. The shape evolution progressed with the regioselective deposition of gold atoms guided by dynamic hotspots, which were strongly localized on the corners of trigonal nanoprisms upon plasmon excitation and, thus, led to CPL dependence.

### 2.2. Chiral Assembly Superstructures of Plasmonic Nanoparticles

#### 2.2.1. Molecular Linker

**Figure 2:**
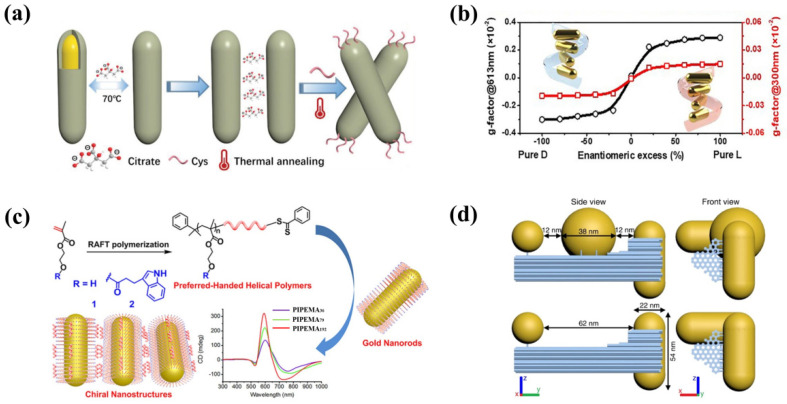
Fabrication of chiral plasmonic superstructures via self-assembly. (**a**) Effects of metal composition on chiroptical activity of rod assemblies [60]. Copyright 2020, Wiley. (**b**) Demonstration of majority-rule in chiral assemblies of AuNRs driven by Cys molecules-mediated chiral environments [61]. Copyright 2021, American Chemical Society. (**c**) Chiral assemblies of AuNRs linked by helical polymers of PIPEMAx and their PCD responses [24]. Copyright 2019, American Chemical Society. (**d**) DNA origami-guided precise positioning of AuNPs [73]. Copyright 2021, Springer Nature.

Apart from discrete chiral gold NPs, more research studies have been conducted on forming chiral assemblies using achiral gold NPs due to the wide availability of gold NPs with different sizes and morphologies. Among various gold NPs, AuNRs again catch the favor in researchers’ eyes. As mentioned in the Introduction, chiral molecules play key roles in inducing plasmonic CD responses in the chiral assembly structures of plasmonic NPs [22,23,60,61]. Our group developed a simple method to form chiral oligomers of AuNRs by adding chiral thiols on the achiral assemblies of AuNRs [19]. Upon thiol adsorption, the formation of local chiral fields drove rod twisting within the assemblies and led to the formation of chiral superstructures. The role of chiral force was demonstrated by CPL-modulated CD responses of the chiral AuNR dimer [23]. Due to the energy demand of rod twisting, proper temperature elevation was found to promote the formation of chiral assemblies and produced a PCD temperature amplification effect. As thiol–metal interactions are related to the metal’s composition, we further investigated the effect of shell compositions by employing AuNR@Au_x_Ag_1−x_ core–shell monomers as building blocks [60]. Very interestingly, for AuNR@Ag assemblies upon adding Cys, they showed opposite CD signs and different temperature behaviors compared to AuNR@Au counterparts (Figure 2a). At a Cys incubation temperature of 30 °C, AuNR@Ag assemblies showed an asymmetric factor of 0.031, which is much larger than −1.1 × 10^−3^ of their AuNR@Au counterparts. In contrast, when raising Cys incubation temperatures to 60 °C, Ag-based assemblies had no observable CD signals, whereas the g factor of AuNR@Au assemblies increased to −0.007. Such quite distinct behaviors were ascribed to the dependence of the Cys chiral network on exposed lattice planes of rods and the destruction of the Cys chiral network by the enhanced migration of surface Ag atoms at high temperatures. Apart from the dominant roles of chiral thiol and metal surfaces, Wang et al. found that chiral environments might assist the formation of chiral assemblies [61]. They investigated the effect of CTAB concentrations in rod dispersion media on the chiroptical activities of AuNR oligomers. Chiral AuNR oligomers obtained in 0.5 mM CTAB (Type I) exhibited weaker PCD responses compared to their counterparts dispersed in 2 mM CTAB (Type II). In addition, a nonlinear PCD amplification with enantiomeric excess (ee) was observed in Type II system. The twin “majority-rules” effects, observed both in CTAB-citrate-Cys molecular environments and in Type II plasmonic superstructures suggested that the molecular environment might act as a dynamic chiral template to assist the formation of chiral assemblies (Figure 2b). In contrast, a linear PCD change with ee was found for Type I plasmonic superstructures, indicating the absence of chiral molecular environment at low CTAB amounts. Besides small chiral molecules and bio-macromolecules, synthetic chiral polymers show promises in the construction of chiral plasmonic superstructures. For instance, using reversible addition–fragmentation chain transfer polymerization (RAFT), Liu et al. synthesized hydroxyethyl methacrylate-3-indole propionate (PIPEMA_x_) molecules with different polymerization degrees (x). By using them as linkers for the AuNRs, chiral assemblies were obtained (Figure 2c). Moreover, the magnitude of chiroptical signals can be modulated by changing the polymerization degree [24]. TEM images of the chiral assemblies indicated the tilting of AuNRs within the assemblies as the origin of PCD responses. 

#### 2.2.2. Chiral Templates

Among various chiral templates [62], DNA origami, with precise structure designs based on DNA molecules, provides an unprecedented platform to anchor the positions of molecules and nano-objects precisely [63,64,65,66,67,68,69,70,71,72]. For the majority of chiral templates, NPs acquire a chiral configuration by following the chiral patterns of the templates. DNA origami can be considered as the most precise chiral templates from its incomparable ability in controlling the spatial arrangement of plasmonic NPs and thus modulating assembly configuration. A good example is recently demonstrated by Liedl and collaborators [73]. They first positioned two AuNRs in a chiral configuration with a distance of 62 nm on a DNA origami structure and then arranged a 38 nm gold nanosphere (AuNS) in between (Figure 2d). They found that the AuNS greatly enhanced CD responses of AuNR chiral dimer. In addition, an additional CD signal occurred at the SPR position of the nanosphere, indicating the transfer and enhancement of chiral fields over distances close to 100 nm.

## 3. Optical Properties of Chiral Plasmonic Nanomaterials

**Figure 3:**
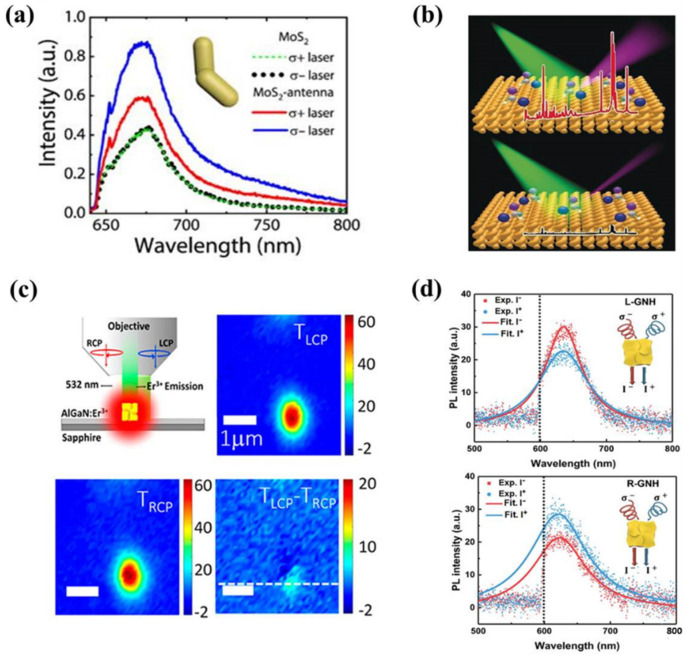
Novel optical properties of chiral plasmonic nanomaterials. (**a**) Valley-polarized emission of monolayer MoS_2_ sandwiched between a chiral AuNR dimer. Reproduced with permission [78]. Copyright 2020, Wiley. (**b**) Enantiomer discrimination based on the SERS-ChA method [80]. Copyright 2020, Wiley. (**c**) Experimental thermal images of chiral helicoid under LCP and RCP illuminations [77]. Copyright 2020, American Chemical Society. (**d**) Chiral PL features of L- and R-GNHs. Reproduced with permission [79]. Copyright 2021, Wiley.

With the rapid development of chiral plasmonic nanostructures/superstructures, further study of their chirality-related optical properties become possible, such as chiral-field-steered emissions and surface-enhanced Raman scattering (SERS) [74,75], photothermal chirality [76,77], and chiral photoluminescence (PL) from chiral structures themselves [78,79] to name a few (Figure 3). Using the combination of chiral plasmonic antenna and monolayer transition metal dichalcogenides, Lu et al. demonstrated the huge modulation of the valley-polarized PL of monolayer MoS_2_ located in a nanogap of two AuNRs in a chiral configuration (Figure 3a) [78]. CPL-modulated emission can be ascribed to the regulation of the valley-dependent excitons by chiral antennas. Compared to field-enhanced emission, SERS exhibits a stronger dependence on local electromagnetic fields. Recently, Che et al. proposed a new strategy to discriminate enantiomers by combining chiral plasmonic nanostructures with the traditional SERS method [80]. They introduced a SERS-chiral anisotropy (SERS-ChA) effect, which was based on enantiomer-selective SERS enhancements on a chiral plasmonic surface and thus provided high enantiomeric sensitivity (Figure 3b). A new parameter g_SERS-ChA_ was defined as 2(*I*_S_ − *I*_R_)/ (*I*_S_ + *I*_R_), where (*I*_S_ − *I*_R_) denoted SERS intensities of S- and R- enantiomers, respectively. Obviously, a g_SERS-ChA_ absolute value of 2 suggested a complete discrimination of enantiomers. Compared with surface-enhanced Raman optical activities (SEROAs) [81], SERS-ChA has more advantages in enantiomeric resolutions and method compatibility with normal Raman spectrometers.

As is well-known, the large absorption cross-section of plasmonic NPs renders them as good photothermal materials. After forming chiral structures, a novel concept of photothermal chirality was suggested by Govorov et al. [76], which reflected the photothermal temperature difference from chiral meta-structures upon irradiation with LCP and RCP lights. Using gold nanohelicoids (GNHs) prepared by seed-mediated chiral growth, they demonstrated this effect experimentally (Figure 3c) [77]. By placing GNHs on AlGaN: Er^3+^ film and using the luminescence of Er^3+^ as temperature probes, they obtained thermal maps upon irradiation under 532 nm CPL. The GNHs showed a temperature difference of ~6 K between LCP and RCP laser irradiations. Again, using GNHs prepared by the procedure suggested by Nam et al., they reported chiral photoluminescence (PL) from GNHs themselves [79]. Despite opposite CD features of the left-handed GNH (L-GNH) and right-handed GNH (R-GNH), the GNH enantiomers also exhibited circular polarization-regulated chiral PL signals (Figure 3d), which originated from differential absorptions of the GNH to CPL and enhanced localized electromagnetic fields overlapping at both excitation and emission spectral regions. In all, these chirality-related optical properties provide more means for bio-detection and imaging.

## 4. Biomedical Applications

### 4.1. Biosensing Based on Plasmonic Chiral Responses

Due to large chiroptical activities, various bio-sensors based on chiral plasmonic nanomaterials have been demonstrated (Table 1), showing great potentials in enantioselective sensing and early disease diagnosis. 

**Figure 4:**
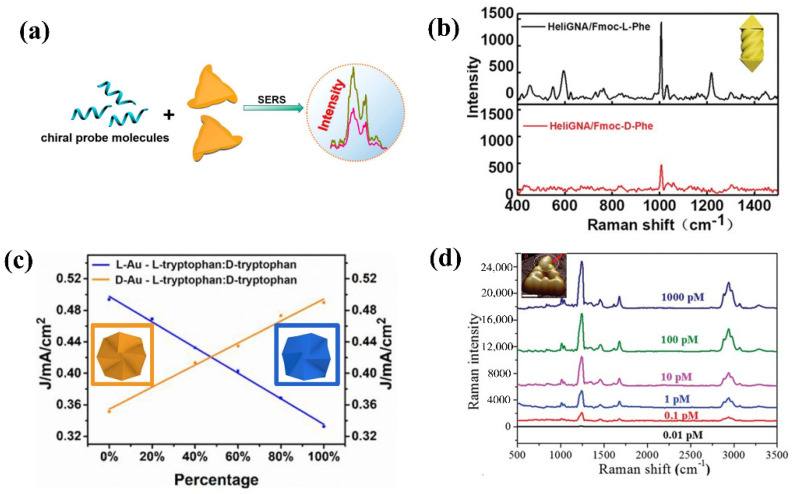
Biosensing based on discrete chiral plasmonic nanostructures. (**a**) Schematic illustration of D-Au and L-Au propellers as SERS substrates for the detection and discrimination of chiral molecules [82]. Copyright 2020, American Chemical Society. (**b**) HeliGNAs as SERS substrates for recognition of Fmoc-L/D-Phe [83]. Copyright 2021, Wiley. (**c**) Chiral Au nanocrystals-modified electrodes for tryptophan detection [84]. Copyright 2022, American Chemical Society. (**d**) SERS detection of Aβ fibrils on chiral Pt@Au TNR substrate [85]. Scale bar, 50 nm. Copyright 2021, Wiley.

Using chiral Au nanotriangles (C-AuNTs) with a propeller shape as a SERS substrate, Ma et al. demonstrated the detection and discrimination of chiral biomolecules, such as L-Dopa, doxorubicin (DOX), and L/D-carnitine [82]. L-Cys-mediated L-Au propellers gave opposite plasmonic CD signals to their D-Au counterparts mediated by D-Cys molecules (Figure 4a). L-Dopa molecules dispersed on the substrate composed of D-Au propellers showed a 5-fold stronger SERS intensity than those dispersed on L-Au counterparts. Density functional theory (DFT) calculations indicated that partial chiral recognitions originated from the difference in the binding strength of L-Cys and D-Cys with the detected chiral molecules, i.e., D-type chiral molecules have a stronger molecular interaction with L-type chiral molecules. However, achiral AuNTs post-modified with L-Cys or D-Cys had no such chiral differentiation effects. Chiral Au propellers themselves may play a role. 

Using L-selenocystine (L-SeCys_2_) as a chiral inducer and AuNRs as seeds, Liu et al. synthesized chiral Au nanoarrows (GNAs). It was found that the pre-irradiation of the L-SeCys_2_ solution with a UV light played an important role in the formation of helical grooves. In the absence of pre-irradiation, GNAs showed no observable helical grooves and produced weak PCD responses. In contrast, adding pre-irradiated L-SeCys_2_, the obtained GNAs exhibited obvious helical grooves and produced 10-fold stronger PCD responses compared to GNAs with an opposite sign [83]. The generation of selenyl radicals upon photo-irradiation was suggested as crucial for the formation of helical grooves. Raman signals of Fmoc-L-phenylalanine (Fmoc-L-Phe) on both GNAs and HeliGNAs were stronger than those of Fmoc-D-Phe (Figure 4b). Considering the opposite PCD signals of GNAs and HeliGNAs, they proposed that the specific interaction between chiral ligands and enantiomers was responsible for the observed chiral discrimination. 

More recently, Niu et al. demonstrated the synthesis of chiral Au trisoctahedral Au NPs with homochiral facets by using binary surfactants to control the overgrowth process [84]. L- and D-Au with $\{12\bar{58}{\}}^{R}$and $\{8\bar{51}2{\}}^{S}$ high Miller index facets are obtained from L- and D-Cys-mediated growth, respectively. Achiral Au NPs were obtained with racemic Cys. Chiral-nanocrystal-modified electrodes were able to show enantioselective differential pulse voltammograms (DPVs). L-Au showed a higher peak current for L-tryptophan while D-Au exhibited a better response for D-tryptophan. A linear relationship was obtained between the DPV peak current vs. ee value (Figure 4c). Enantioselective interactions between tryptophan enantiomers and homochiral surfaces were suggested to be the source of chiral discrimination. 

Using platinum triangular nanorings (Pt TNRs) as seeds, Xu et al. synthesized L/D-Pt@Au TNRs under the guide of L-/D- glutathione, respectively, which had obvious chiral plasmonic responses and showed SERS-ChA effects [85]. D-Pt@Au TNRs produced stronger Raman signals in detecting Aβ_42_ monomers and fibrils with the limit of detection (LOD) of 45 × 10^−15^ M and 4 × 10^−15^ M, respectively (Figure 4d). Compared to L-Pt@Au TNRs, larger binding constants between D-Pt@Au TNRs and proteins were obtained using isothermal titration calorimetry measurements. Furthermore, D-Pt@Au TNRs were able to detect Aβ_42_ proteins in Alzheimer’s disease (AD) patients at the pictogram level.

In comparison with discrete chiral plasmonic nanostructures, the dynamically tunable nature of plasmonic superstructures facilitates higher detection sensitivities based on PCD signals. Chiral assemblies have been widely explored for the detection of various bio-related substances, such as metal ions of Hg^2+^ [26,86], Ag^+^ [87], Pb^2+^ [88], Zn^2+^ [89], and Cu^2+^ [89,90]; DNA/RNA [91,92,93,94,95]; amino acids/peptides [96,97,98]; proteins [99,100]; antigens [101,102]; and other biomarker molecules [103,104,105].

**Figure 5:**
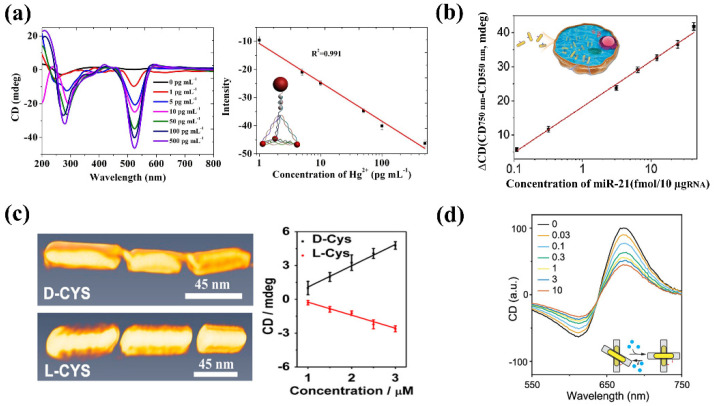
Chiral plasmonic superstructures for biosensing based on PCD changes induced by adding analytes. (**a**) Plasmonic HPs for ultra-sensitive detection of Hg^2+^ [86]. Copyright 2015, Elsevier. (**b**) In vitro detection of miRNA using chiral AuNR dimers [91]. Copyright 2018, Wiley. (**c**) Chiral Cys-driven end-to-end assembly of AuNRs for their discrimination [96]. Copyright 2015, American Chemical Society. (**d**) Aptamer molecular lock-based chiral plasmonic biosensor for adenosine detection [106]. Copyright 2018, American Chemical Society.

Due to the high sensitivity of plasmonic responses on chiral assembly structure, PCD signals are often used as the detection signal (Figure 5). Zhang et al. first formed an Au trimer using 10 nm Au nanospheres via DNA strand hybridizations and then attached a 25 nm gold nanosphere modified with T-rich DNA sequences to the Au trimer. The obtained heteropyramids (HPs) exhibited obvious chiroptical activities [86]. Via T-Hg^2+^-T interactions, the addition of Hg^2+^ affected the formation of HPs and, thus, their PCD responses. The selective detection of Hg^2+^ was achieved in the range of 1–500 pg mL^−1^ with an LOD of 0.2 pg mL^−1^, which was lower than 1 pg mL^−1^ safety requirements for Hg^2+^ in water (Figure 5a). Based on the microRNA-induced side-by-side self-assembly of AuNRs, which were modified with specific DNA sequences, Xu et al. demonstrated the detection of microRNA -21 (miRNA-21) in living cells [91]. The obtained chiral AuNR dimers had remarkable PCD signals in the visible spectral region. As the amount of miRNA-21 increased, CD signal intensities at 750 nm and 550 nm correspondingly enhanced, leading to an ultrasensitive mi-RNA detection based on the CD signal. The ΔCD (CD_750 nm_−CD_550 nm_) value showed a good linear relationship with miRNA-21 amount at the concentration range of 0.11–42.23 fmol/10 μg _RNA_ with an LOD of 0.081 fmol/10 μg _RNA_ (Figure 5b). Liu et al. reported the application of chiral plasmonic structures in amino acid or peptide detection [96]. Cys or GSH can preferentially adsorb at AuNR ends and leads to AuNR assemblies in end-to-end (EE) fashion. The obtained EE assemblies showed chirality and dose-dependent PCD responses and could be used for enantiomeric discrimination/detection (Figure 5c). For L-Cys/D-Cys, the detection range was 1–3 μM with an LOD of 0.8 μM. For GSH, the detection range was 1–5 μM with an LOD of 1.93 μM. Based on the precise positioning ability of DNA origami, Kuzyk et al. immobilized two AuNRs on the origami and used their plasmonic CD signals for the sensitive detection of adenosine (Figure 5d) [106]. In the presence of adenosine and via the formation of aptamer–adenosine complexes, aptamer molecular locks can affect the chiral configuration of AuNR dimers via the DNA origami structure and, thus, cause changes in PCD signals. As a demonstration, they used a double stranded lock with 9 bp hybridization length (ds9-nanosensor) and exhibited the detection of adenosine with an LOD of approximately 20 μM.

### 4.2. Bio-Imaging of Chiral Plasmonic Nanomaterials

With the rapid progress in fabricating chiral plasmonic nanomaterials using chemical methods, plasmonic chirality-related properties have been introduced to the biological imaging field [107,108,109]. Hybridizing with other functional materials is often adopted to provide multiple imaging modalities. For instance, compared with the traditional down-conversion luminescence materials, upconversion nanoparticles (UCNPs) have a narrower band emission, longer fluorescence lifetime, and low toxicity with large potentials for biomedical applications. Kuang et al. fabricated chiral AuNR-UCNP tetramers with a propeller-like geometry based on DNA-driven self-assembly [110]. The obtained chiral tetramers showed not only strong chiroptical activity but also greatly enhanced UCNP luminescence for in vitro fluorescence imaging. By controlling the distance between the UCNP and rods, a 21.3-fold luminescence increase was obtained. The intensity evolution profile of upconversion luminescence matched tetramer formation times, which were determined by DNA hybridization.

**Figure 6:**
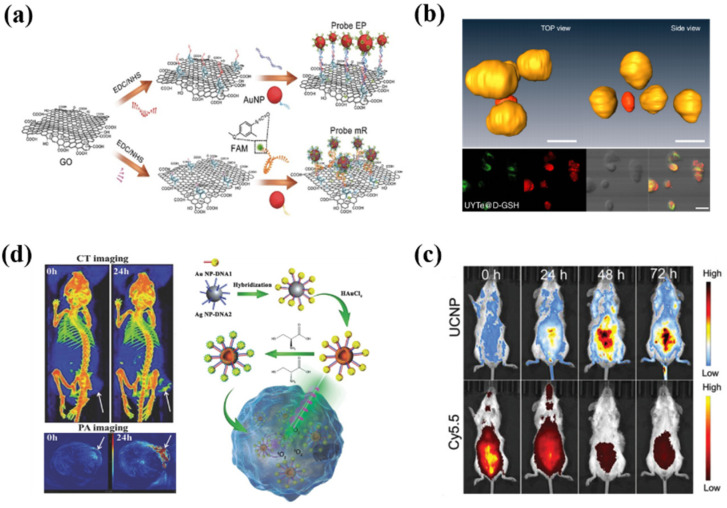
Bio-imaging based on chiral plasmonic nanomaterials or their hybrid nanostructures with other functional materials. (**a**) Raman and confocal images of MCF-7, HeLa, and primary uterine fibroblast (PCS-460-010) cells upon exposure to GO-AuNP chiral hybrid assemblies [111]. Copyright 2017, Wiley. (**b**) In vitro luminescence imaging of chiral ligand-dependent autophagy induced by UYTe [112]. scale bar for TEM images, 20 nm. scale bar for confocal images, 200 nm. Copyright 2018, Springer Nature. (**c**) In vivo tracking the apoptosis process using fluorescence imaging of the UAuTe tetrahedron in senescence-accelerated mice (SAMP8) under different conditions [113]. Copyright 2020, Wiley. (**d**) Schematic of self-assembled plasmonic shell–satellite nanostructure as a chiral PDT agent and its CT and PA bi-modal imaging pictures [114]. Copyright 2017, Wiley.

Using a DNA-hybridization-driven co-assembly of graphene oxide and AuNPs (Figure 6a), Kuang et al. fabricated hybrid probe Ep and hybrid probe mR and demonstrated their applications for in vitro imaging and the detection of EpCAM and miR-21 using different optical modalities [111]. Upon the exposure of chiral hybrid probes to different cells, Raman and fluorescence images indicated high levels of EpCAM and miR-21 in MCF-7 cells, medium levels in HeLa cells, and the lowest levels in primary uterine fibroblast cells. Moreover, using the DNA hybridization strategy, the same group fabricated a chiral nanoprobe for the detection of metal ions [89]. UCNPs were attached on AuNR@Pt chiral dimers in a satellite assembly structure. Both the formation of rod dimer and the linking of UCNPs with the rod dimer were based on the hybridization of M^2+^ (Mg^2+^/Zn^2+^/Cu^2+^)-specific DNAzymes, M^2+^-protected DNA strands, and M^2+^-substrate DNA strands. Using the temperature difference caused by the photothermal chirality effect, upon exposure to the preferred CPL, the protected DAN strand is separated from the probe. In the presence of M^2+^, the M^2+^ -specific DNAzyme activates the corresponding substrate DNA for cleaving and thus restores luminescence signals for M^2+^ sensing.

Chiral ligands on the surface of chiral plasmonic NPs may induce biological effects, which could be revealed by biological imaging. As demonstrated by the Kuang group, for yolk-shell NP tetramers with a central upconversion NP as the yolk and four plasmonic NPs as shells (UYTe), after modifying with L/D-GSH, UYTe@GSH produced chiral ligand-dependent autophagy-inducing activities [112]. As shown in Figure 6b, UYTe@D-GSH induced the strongest green fluorescence signal of autophagy, which was even larger than the positive control of rapamycin (RaPa). In contrast, UYTe@L-GSH and UYTe led to weaker autophagy. After injection into the mice model, in vivo imaging showed similar results. Encouraged by such structural designs, Kuang et al. further prepared a NIR-responsive UAuTe tetrahedron (Figure 6c) [113]. After surface modification and Granzyme B loading, the tetrahedra selectively targeted senescent cells and induced apoptosis upon NIR light exposure due to tetrahedron disassembly and drug release. From in vivo fluorescence images, it took 24 h for the drug to arrive at the targeted cells and another 48 h for Granzyme B to induce apoptosis in senescent cells. 

Biological imaging-guided disease therapy has also been demonstrated in chiral plasmonic assemblies. By forming chiral shell-satellite (SS) gold nanostructure, Xu et al. suggested a chiral photodynamic therapy (PDT) agent [114]. Due to the strong chiroptical activity, the chiral PDT showed CPL-dependent ROS generation. The SS-D-Cys agent exhibited stronger PDT efficacies after injection into tumor-bearing mice. In combination with their X-ray CT and photoacoustic (PA) imaging abilities (Figure 6d), a bimodal imaging-guided treatment is expected.

### 4.3. Disease Diagnosis and Treatment

Similarly to the above biosensing strategy, obvious changes in chiroptical responses, induced upon the binding of targeted disease markers to chiral plasmonic nanomaterials, are used as detection signals to achieve sensitive disease diagnoses. In addition, the combination of chiral plasmonic nanomaterials with various imaging modalities is applied to image-guided diagnosis and treatment. Furthermore, CPL-dependent light absorption makes light-modulated drug release and therapy possible.

**Figure 7:**
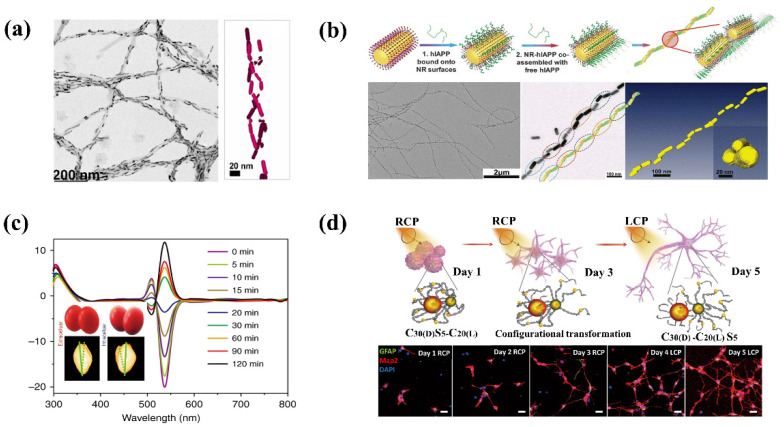
Chiral plasmonic nanomaterials for disease diagnosis based on changes of the PCD signal. (**a**) TEM images showing chiral arrangement of AuNRs on α-synuclein protofibrils and incubation-dependent formation of chiral superstructures [115]. Copyright 2018, National Academy of Sciences. (**b**) Nanohelix superstructures via co-assembly of hIAPPs-modified AuNRs and hIAPPs [116]. Copyright 2021, American Association for the Advancement of Science. (**c**) CD spectrum evolution of DNA-linked AuNP dimers upon co-incubation with cells [117]. Copyright 2017, Springer Nature. (**d**) Fox3 mRNA-triggered the conformational transition of NP dimers for the light-polarization modulated differentiation of neural stem cells (NSC) [118]. Scale bars, 20 μm. Copyright 2021, Springer Nature.

The adsorption of achiral plasmonic NPs on protein aggregates is a useful method for forming chiral plasmonic superstructures. In return, it also enables the diagnosis and prediction of protein-related diseases using chiral plasmonic signals. Liz-Marzán et al. reported such a diagnostic platform for neurodegenerative diseases, such as Alzheimer’s disease (AD), upon the adsorption of AuNRs on α-synuclein protofibrils [115]. The chiral arrangement of AuNRs along the protein fibrils was witnessed from TEM images and was further substantiated by helical configurations via image reconstruction (Figure 7a). During an 80 min co-incubation of AuNRs with α-synuclein protofibrils, PCD signals gradually enhanced with time. No CD responses were observed from the co-incubation of AuNRs with α-synuclein monomers under the same conditions, indicating that AuNRs showed no significant interactions with α-synuclein monomers. In contrast, helical structures on amyloid protofibrils generated by α-synuclein aggregation resulted in the chiral arrangement of AuNRs, which was the source of chiroptical activity. The sensitive PCD response allowed the detection of α-synuclein protofibrils at nanomolar levels, providing a potentially effective platform for the diagnosis of diseases associated with amyloids. Furthermore, using the co-assembly of human islet amyloid polypeptides (hIAPPs)-modified AuNRs and hIAPPs, Liu et al. obtained chiral plasmonic superstructures with CD signals up to 2 deg (g-factor of 0.12) due to the long-range order of assembly structure [116]. It showed a 4600-fold PCD amplification compared to individual NRs modified with hIAPPs. The hIAPP peptide is a hallmark pathological feature of type II diabetes and is also an important biomarker for pancreatic cancer. The substantial PCD signal promises the early diagnosis of related diseases and effective drug screening. 

Xu et al. prepared a chiral AuNP dimer linked by DNA molecules and investigated their cellular behaviors [117]. Upon transmembrane transports in Hela cells, the chiroptical signal of NP dimers experienced a sign reversal (Figure 7c). TEM tomography images indicated a conformation inversion of the AuNP dimer upon entering the cell. The change in electrostatic force was suggested to result in a new equilibrium configuration. After loading photosensitizer PpIX, they exhibited light-polarization-dependent ROS generation and PDT efficacy. At 0.5 μM PpIX, the viability of HeLa cells upon 532 nm LCP irradiation 30 min was approximately 3-fold low than that irradiated with linearly polarized (LP) light or RCP light.

Recently, Xu et al. further found that optically active chiral nanostructures can promote the differentiation of neural stem cells under CPL, thereby improving cognitive impairments in AD mice [118]. Chiral plasmonic nanostructures were formed via DNA hybridization. AuNPs at 5 nm as satellite particles were first bound with D-Cys-functionalized 30 nm AuNP (C_30(D)_) and then bound with L-Cys-functionalized 20 nm AuNP, forming C_30(D)_ S_5_ -C_20(L)_. Interestingly, when the DNA sequence linking 5 nm AuNP and 30 nm AuNP can complement the Fox3 mRNA sequence expressed when neural stem cells differentiate into neurons, the S_5_ NPs will move from C_30(D)_ to C_20(L)_ and transform into C_30(D)_ -C_20(L)_ S_5_ with a reversal of the chiral optical signal (Figure 7d). It was shown that CPL-differentiated NSC cells can be used for the treatment and amelioration of AD in the mouse model. During a 90-day observation period after treatment, two markers of AD, amyloid-β (Aβ) and hyperphosphorylated tau (p-tau) protein, were both reduced by more than 70% in cerebrospinal fluids extracted from AD mice, and there was a significant improvement in the cognitive performance of AD mice. 

## 5. Conclusions and Perspective

We witnessed a rapid development in plasmon-based chiral metamaterials over the past decade. Thanks to the progress of wet-chemical synthesis, discrete plasmonic NPs/nanostructures with clear 3D chiral morphology and large plasmonic chiroptical activity now can be obtained via seeded-mediated growth. It is suggested that chiral thiol-based strong ligands stabilize high-index chiral facets and lead to the formation of chiral morphology. Up until now, the reported most successful chiral ligands are small chiral cysteine. Screening efficient chirality-inducing thiols via various theoretical simulations may promote the selection and rational design of proper chiral ligands. Apart from strong thiol ligands, non-valence chiral surfactants also demonstrated the induction of chiral growth. Surface chiral templates are thought to be responsible. The detailed mechanism needs further study in order to achieve more the precise control of chiral morphologies. In addition, the effects of growth kinetics have yet been systematically investigated. Furthermore, in combination with CPL during chiral growth, obviously improved chiroptical responses indicate that light-modulated chiral growth is beginning to emerge and may open a new road for chiral synthesis in the future. As demonstrated herein, most research studies employ gold, and the successful extension of this growth strategy to other plasmonic metals is needed. In comparison with top-down methods, the advantage of chemical synthesis is the control of the chiral gap, which may provide a unique chiral microenvironment for asymmetric catalysis and enantiomeric separation. 

For chiral plasmonic assemblies/superstructures, the bottom-up assembly of achiral plasmonic nanoparticles is widely employed either by using chiral molecules as linkers, providing chiral driving forces to form chiral assemblies, or by forming asymmetric adsorption configuration on chiral templates/media. Although strong plasmonic optical activities are demonstrated, the precise arrangement of achiral NPs on chiral templates is still facing challenges. Majority-rule and sergeant-and-soldier effects in supramolecular chirality are also demonstrated in the chiral assemblies of AuNRs under specific conditions, which may indicate their extension to the fabrication of chiral plasmonic superstructures. We believe that chirality rules found in supramolecular systems may be borrowed can benefit chiral plasmonic superstructures. Still, mechanisms and the precise maneuvers of cross-length chirality transfer and amplification need further exploration [22,61,73,119,120].

Obviously, the introduction of chiral plasmonic nanostructures/superstructures will enhance the chiroptical properties of other chiral materials, such as CD, CPL, and SEROA. Among them, the SERS-ChA effect is a surprise, which could realize the enantiomeric discrimination via normal Raman spectrometer in combination with a chiral plasmonic substrate. Despite these, the chiroptical properties themselves improved, such as chiral photoluminescence and photothermal circular dichroism.

**Scheme 1:**
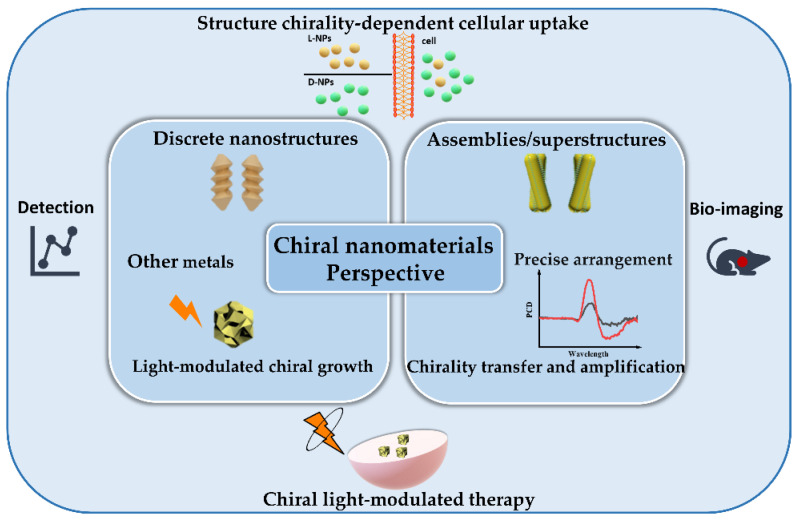
Future perspectives of chiral nanomaterials for biomedical applications.

Hybridizing chiral plasmonic NPs with other nanomaterials (such as magnetic NPs and semiconductor quantum dots) may present a future research direction. At one hand, it can overcome the shortcomings of plasmonic nanomaterials themselves (poor photoluminescence efficiency and low separation efficiency of photo-generated carriers). On the other hand, it can enrich functionalities and broaden application ranges. The Kuang group demonstrated pioneering work by using hybrid chiral assemblies in disease diagnoses and treatments. High detection sensitivities have been demonstrated. However, considering the complexity of biological systems, the advantage of sensitivity may be compromised by low reproducibility. Some interesting observations, such as structure chirality-dependent cellular uptake and chiral light-modulated therapy, have been demonstrated. Further mechanism investigations and buildup of structure–activity relationships are needed before these new ideas and options can be applied in disease diagnoses and therapy. Future perspectives of chiral nanomaterials are summarized in Scheme 1. In all, chiral plasmonic nanomaterials have great potentials in biomedical fields.

## Figures and Tables

**Table 1:** AuNP-based chiral nanomaterials for analyte detection.

Chiral Nanomaterials	Target Analyte	Response Signals	Ref.
Au nanotriangles	chiral biomolecules	SERS	[82]
Au nanoarrows	Fmoc-L/D-phenylalanine	SERS	[83]
Au trisoctahedral NPs	L/D-tryptophan	Differential Pulse Voltammograms	[84]
Pt@Au triangular nanorings	Aβ_42_ monomers and fibrils	SERS	[85]
AuNR assemblies	Hg^2+^	PCD	[26]
AuNPs heteropyramids	Hg^2+^	PCD	[86]
AuNPs heterodimers	Ag^+^	PCD	[87]
AgNPs dimers	Pb^2+^	PCD	[88]
AuNR@Pt dimers and UCNPs	Cu^2+^, Mg^2+^, Zn^2+^	Fluorescence intensity	[89]
AuNR assemblies	Cu^2+^	PCD	[90]
AuNR@PS-PAA dimers	miRNA-21	PCD	[91]
AuNR assemblies	DNA	PCD	[92]
AuNP pyramids	DNA	PCD	[93]
AuNR dimers	microRNA	PCD	[94]
Two-layer core-satellite gold nanostructures	microRNA	PCD/SERS	[95]
AuNR assemblies	Cysteine	PCD	[96]
AuNP pyramids	DNaseⅠ	PCD	[97]
AuNP dimers	Cysteine	PCD	[98]
AuNP dimers	alpha-fetoprotein	PCD	[99]
AuNR dimers	Thrombin	PCD	[100]
AuNP and Ag NP heterodimers	Prostate-specific antigen (PSA)	PCD	[101]
Au @Ag NR dimers	PSA	PCD	[102]
AuNP heterodimers	ATP	PCD	[103]
DNA-driven AuNP heterodimers	Intracellular telomerase	PCD	[104]
DNA origami-based AuNR dimers	small molecules, living tumor cells	PCD	[105]
DNA origami-based AuNR dimers	adenosine	PCD	[106]

## References

[1] Kelvin W.T. (1894). The Molecular Tactics of a Crystal.

[2] Quidant R., Kreuzer M. (2010). Plasmons offer a helping hand. Nat. Nanotechnol..

[3] Orfanidis S.J. (2002). Electromagnetic Waves and Antennas.

[4] Hao C., Gao Y., Wu D., Li S., Xu L., Wu X., Guo J., Sun M., Li X., Xu C. (2019). Tailoring chiroptical activity of iron disulfide quantum dot hydrogels with circularly polarized light. Adv. Mater..

[5] Xia Y., Zhou Y., Tang Z. (2011). Chiral inorganic nanoparticles: Origin, optical properties and bioapplications. Nanoscale.

[6] Guo Y., Zhao X., Long T., Lin M., Liu Z., Huang C. (2015). Histidine-mediated synthesis of chiral fluorescence gold nanoclusters: Insight into the origin of nanoscale chirality. RSC Adv..

[7] Wang X., Tang Z. (2017). Circular dichroism studies on plasmonic nanostructures. Small.

[8] Yang L., Liu J., Sun P., Ni Z., Ma Y., Huang Z. (2020). Chiral ligand-free, optically active nanoparticles inherently composed of chiral lattices at the atomic scale. Small.

[9] Wang Y., Xu J., Wang Y., Chen H. (2013). Emerging chirality in nanoscience. Chem. Soc. Rev..

[10] Zhu Z., Guo J., Liu W., Li Z., Han B., Zhang W., Tang Z. (2013). Controllable optical activity of gold nanorod and chiral quantum dot assemblies. Angew. Chem. Int. Ed. Engl..

[11] Zheng G., He J., Kumar V., Wang S., Pastoriza-Santos I., Perez-Juste J., Liz-Marzan L.M., Wong K.Y. (2021). Discrete metal nanoparticles with plasmonic chirality. Chem. Soc. Rev..

[12] Govorov A.O., Fan Z., Hernandez P., Slocik J.M., Naik R.R. (2010). Theory of circular dichroism of nanomaterials comprising chiral molecules and nanocrystals: Plasmon enhancement, dipole interactions, and dielectric effects. Nano Lett..

[13] Slocik J.M., Govorov A.O., Naik R.R. (2011). Plasmonic circular dichroism of peptide-functionalized gold nanoparticles. Nano Lett..

[14] Hao C., Xu L., Ma W., Wu X., Wang L., Kuang H., Xu C. (2015). Unusual circularly polarized photocatalytic activity in nanogapped gold-silver chiroplasmonic nanostructures. Adv. Funct. Mater..

[15] Lee H.E., Ahn H.Y., Mun J., Lee Y.Y., Kim M., Cho N.H., Chang K., Kim W.S., Rho J., Nam K.T. (2018). Amino-acid- and peptide-directed synthesis of chiral plasmonic gold nanoparticles. Nature.

[16] Lv J., Gao X., Han B., Zhu Y., Hou K., Tang Z. (2022). Self-assembled inorganic chiral superstructures. Nat. Rev. Chem..

[17] Han B., Zhu Z., Li Z., Zhang W., Tang Z. (2014). Conformation modulated optical activity enhancement in chiral cysteine and au nanorod assemblies. J. Am. Chem. Soc..

[18] Han B., Shi L., Gao X., Guo J., Hou K., Zheng Y., Tang Z. (2015). Ultra-stable silica-coated chiral au-nanorod assemblies: Core–shell nanostructures with enhanced chiroptical properties. Nano Res..

[19] Yan J., Hou S., Ji Y., Wu X. (2016). Heat-enhanced symmetry breaking in dynamic gold nanorod oligomers: The importance of interface control. Nanoscale.

[20] Zhai D., Wang P., Wang R.Y., Tian X., Ji Y., Zhao W., Wang L., Wei H., Wu X., Zhang X. (2015). Plasmonic polymers with strong chiroptical response for sensing molecular chirality. Nanoscale.

[21] Bao Z.Y., Dai J., Zhang Q., Ho K.H., Li S., Chan C.H., Zhang W., Lei D.Y. (2018). Geometric modulation of induced plasmonic circular dichroism in nanoparticle assemblies based on backaction and field enhancement. Nanoscale.

[22] Hu Z., Meng D., Lin F., Zhu X., Fang Z., Wu X. (2019). Plasmonic circular dichroism of gold nanoparticle based nanostructures. Adv. Opt. Mater..

[23] Zhao W., Zhang W., Wang R.Y., Ji Y., Wu X., Zhang X. (2019). Photocontrollable chiral switching and selection in self-assembled plasmonic nanostructure. Adv. Funct. Mater..

[24] Cheng G., Xu D., Lu Z., Liu K. (2019). Chiral self-assembly of nanoparticles induced by polymers synthesized via reversible addition-fragmentation chain transfer polymerization. ACS Nano.

[25] Yan W., Xu L., Xu C., Ma W., Kuang H., Wang L., Kotov N.A. (2012). Self-assembly of chiral nanoparticle pyramids with strong r/s optical activity. J. Am. Chem. Soc..

[26] Zhu Y., Xu L., Ma W., Xu Z., Kuang H., Wang L., Xu C. (2012). A one-step homogeneous plasmonic circular dichroism detection of aqueous mercury ions using nucleic acid functionalized gold nanorods. Chem. Commun..

[27] Wu X., Xu L., Ma W., Liu L., Kuang H., Kotov N.A., Xu C. (2016). Propeller-like nanorod-upconversion nanoparticle assemblies with intense chiroptical activity and luminescence enhancement in aqueous phase. Adv. Mater..

[28] Dominguez-Medina S., Kisley L., Tauzin L.J., Hoggard A., Shuang B., Indrasekara A.S., Chen S., Wang L.Y., Derry P.J., Liopo A. (2016). Adsorption and unfolding of a single protein triggers nanoparticle aggregation. ACS Nano.

[29] Lu J., Chang Y.X., Zhang N.N., Wei Y., Li A.J., Tai J., Xue Y., Wang Z.Y., Yang Y., Zhao L. (2017). Chiral plasmonic nanochains via the self-assembly of gold nanorods and helical glutathione oligomers facilitated by cetyltrimethylammonium bromide micelles. ACS Nano.

[30] Shinmori H., Mochizuki C. (2017). Strong chiroptical activity from achiral gold nanorods assembled with proteins. Chem. Commun..

[31] Wang Z.Y., Zhang N.N., Li J.C., Lu J., Zhao L., Fang X.D., Liu K. (2021). Serum albumin guided plasmonic nanoassemblies with opposite chiralities. Soft Matter.

[32] Zhang Q., Hernandez T., Smith K.W., Jebeli S.A.H., Dai A.X., Warning L., Baiyasi R., McCarthy L.A., Guo H., Chen D.-H. (2019). Unraveling the origin of chirality from plasmonic nanoparticle-protein complexes. Science.

[33] Merg A.D., Boatz J.C., Mandal A., Zhao G., Mokashi-Punekar S., Liu C., Wang X., Zhang P., van der Wel P.C.A., Rosi N.L. (2016). Peptide-directed assembly of single-helical gold nanoparticle superstructures exhibiting intense chiroptical activity. J. Am. Chem. Soc..

[34] Liu S., Ma X., Song M., Ji C.Y., Song J., Ji Y., Ma S., Jiang J., Wu X., Li J. (2021). Plasmonic nanosensors with extraordinary sensitivity to molecularly enantioselective recognition at nanoscale interfaces. ACS Nano.

[35] Nemati A., Shadpour S., Querciagrossa L., Li L., Mori T., Gao M., Zannoni C., Hegmann T. (2018). Chirality amplification by desymmetrization of chiral ligand-capped nanoparticles to nanorods quantified in soft condensed matter. Nat. Commun..

[36] Cheng Z., Ma Y., Yang L., Cheng F., Huang Z., Natan A., Li H., Chen Y., Cao D., Huang Z. (2019). Plasmonic-enhanced cholesteric films: Coassembling anisotropic gold nanorods with cellulose nanocrystals. Adv. Opt. Mater..

[37] Nemati A., Shadpour S., Querciagrossa L., Mori T., Zannoni C., Hegmann T. (2019). Highly sensitive, tunable chirality amplification through space visualized for gold nanorods capped with axially chiral binaphthyl derivatives. ACS Nano.

[38] Grzelak D., Tupikowska M., Vila-Liarte D., Beutel D., Bagiński M., Parzyszek S., Góra M., Rockstuhl C., Liz-Marzán L.M., Lewandowski W. (2022). Liquid crystal templated chiral plasmonic films with dynamic tunability and moldability. Adv. Funct. Mater..

[39] Jin X., Jiang J., Liu M. (2016). Reversible plasmonic circular dichroism via hybrid supramolecular gelation of achiral gold nanorods. ACS Nano.

[40] Wang S., Zhang Y., Qin X., Zhang L., Zhang Z., Lu W., Liu M. (2020). Guanosine assembly enabled gold nanorods with dual thermo- and photoswitchable plasmonic chiroptical activity. ACS Nano.

[41] Thomas A.R., Swetha K., Aparna C.K., Ashraf R., Kumar J., Kumar S., Mandal S.S. (2022). Protein fibril assisted chiral assembly of gold nanorods. J. Mater. Chem. B.

[42] Kuzyk A., Schreiber R., Fan Z., Pardatscher G., Roller E.M., Hogele A., Simmel F.C., Govorov A.O., Liedl T. (2012). DNA-based self-assembly of chiral plasmonic nanostructures with tailored optical response. Nature.

[43] Lan X., Liu T., Wang Z., Govorov A.O., Yan H., Liu Y. (2018). DNA-guided plasmonic helix with switchable chirality. J. Am. Chem. Soc..

[44] Ma L., Liu Y., Han C., Movsesyan A., Li P., Li H., Tang P., Yuan Y., Jiang S., Ni W. (2022). DNA-assembled chiral satellite-core nanoparticle superstructures: Two-state chiral interactions from dynamic and static conformations. Nano Lett..

[45] Cao Z., Gao H., Qiu M., Jin W., Deng S., Wong K.Y., Lei D. (2020). Chirality transfer from sub-nanometer biochemical molecules to sub-micrometer plasmonic metastructures: Physiochemical mechanisms, biosensing, and bioimaging opportunities. Adv. Mater..

[46] Fan Y., Ou-Yang S., Zhou D., Wei J., Liao L. (2022). Biological applications of chiral inorganic nanomaterials. Chirality.

[47] Ma W., Xu L., Wang L., Xu C., Kuang H. (2019). Chirality-based biosensors. Adv. Funct. Mater..

[48] Cho N.H., Lee H.E., Ahn H.Y., Lee Y.Y., Im S.W., Kim H., Nam K.T. (2019). Cysteine induced chiral morphology in palladium nanoparticle. Part. Part. Syst. Charact..

[49] Cho N.H., Byun G.H., Lim Y.C., Im S.W., Kim H., Lee H.E., Ahn H.Y., Nam K.T. (2020). Uniform chiral gap synthesis for high dissymmetry factor in single plasmonic gold nanoparticle. ACS Nano.

[50] Kim H., Im S.W., Cho N.H., Seo D.H., Kim R.M., Lim Y.C., Lee H.E., Ahn H.Y., Nam K.T. (2020). Gamma-glutamylcysteine- and cysteinylglycine-directed growth of chiral gold nanoparticles and their crystallographic analysis. Angew. Chem. Int. Ed. Engl..

[51] Lee H.E., Kim R.M., Ahn H.Y., Lee Y.Y., Byun G.H., Im S.W., Mun J., Rho J., Nam K.T. (2020). Cysteine-encoded chirality evolution in plasmonic rhombic dodecahedral gold nanoparticles. Nat. Commun..

[52] Wang S., Zheng L., Chen W., Ji L., Zhang L., Lu W., Fang Z., Guo F., Qi L., Liu M. (2021). Helically grooved gold nanoarrows: Controlled fabrication, superhelix, and transcribed chiroptical switching. CCS Chem..

[53] Zhang N.-N., Sun H.-R., Xue Y., Peng F., Liu K. (2021). Tuning the chiral morphology of gold nanoparticles with oligomeric gold–glutathione complexes. J. Phys. Chem. C.

[54] Zhang N.-N., Sun H.-R., Liu S., Xing Y.-C., Lu J., Peng F., Han C.-L., Wei Z., Sun T., Yang B. (2022). Gold nanoparticle enantiomers and their chiral-morphology dependence of cellular uptake. CCS Chem..

[55] Yan J., Chen Y., Hou S., Chen J., Meng D., Zhang H., Fan H., Ji Y., Wu X. (2017). Fabricating chiroptical starfruit-like au nanoparticles via interface modulation of chiral thiols. Nanoscale.

[56] Zheng G., Bao Z., Perez-Juste J., Du R., Liu W., Dai J., Zhang W., Lee L.Y.S., Wong K.Y. (2018). Tuning the morphology and chiroptical properties of discrete gold nanorods with amino acids. Angew. Chem. Int. Ed. Engl..

[57] Chen J., Gao X., Zheng Q., Liu J., Meng D., Li H., Cai R., Fan H., Ji Y., Wu X. (2021). Bottom-up synthesis of helical plasmonic nanorods and their application in generating circularly polarized luminescence. ACS Nano.

[58] Gonzalez-Rubio G., Mosquera J., Kumar V., Pedrazo-Tardajos A., Llombart P., Solis D.M., Lobato I., Noya E.G., Guerrero-Martinez A., Taboada J.M. (2020). Micelle-directed chiral seeded growth on anisotropic gold nanocrystals. Science.

[59] Xu L., Wang X., Wang W., Sun M., Choi W.J., Kim J.Y., Hao C., Li S., Qu A., Lu M. (2022). Enantiomer-dependent immunological response to chiral nanoparticles. Nature.

[60] Meng D., Chen Y., Ji Y., Shi X., Wang H., Wu X. (2021). Temperature effect of plasmonic circular dichroism in dynamic oligomers of aunr@ag nanorods driven by cysteine: The role of surface atom migration. Adv. Opt. Mater..

[61] Song M., Tong L., Liu S., Zhang Y., Dong J., Ji Y., Guo Y., Wu X., Zhang X., Wang R.Y. (2021). Nonlinear amplification of chirality in self-assembled plasmonic nanostructures. ACS Nano.

[62] Vila-Liarte D., Kotov N.A., Liz-Marzan L.M. (2022). Template-assisted self-assembly of achiral plasmonic nanoparticles into chiral structures. Chem. Sci..

[63] Shen X., Asenjo-Garcia A., Liu Q., Jiang Q., de Abajo F.J.G., Liu N., Ding B. (2013). Three-dimensional plasmonic chiral tetramers assembled by DNA origami. Nano Lett..

[64] Kuzyk A., Schreiber R., Zhang H., Govorov A.O., Liedl T., Liu N. (2014). Reconfigurable 3d plasmonic metamolecules. Nat. Mater..

[65] Lan X., Su Z., Zhou Y., Meyer T., Ke Y., Wang Q., Chiu W., Liu N., Zou S., Yan H. (2017). Programmable supra-assembly of a DNA surface adapter for tunable chiral directional self-assembly of gold nanorods. Angew. Chem. Int. Ed. Engl..

[66] Shen C., Lan X., Zhu C., Zhang W., Wang L., Wang Q. (2017). Spiral patterning of au nanoparticles on au nanorod surface to form chiral aunr@aunp helical superstructures templated by DNA origami. Adv. Mater..

[67] Chen Z., Choi C.K.K., Wang Q. (2018). Origin of the plasmonic chirality of gold nanorod trimers templated by DNA origami. ACS Appl. Mater. Interfaces.

[68] Kneer L.M., Roller E.M., Besteiro L.V., Schreiber R., Govorov A.O., Liedl T. (2018). Circular dichroism of chiral molecules in DNA-assembled plasmonic hotspots. ACS Nano.

[69] Nguyen L., Dass M., Ober M.F., Besteiro L.V., Wang Z.M., Nickel B., Govorov A.O., Liedl T., Heuer-Jungemann A. (2020). Chiral assembly of gold-silver core-shell plasmonic nanorods on DNA origami with strong optical activity. ACS Nano.

[70] Wang P., Huh J.H., Park H., Yang D., Zhang Y., Zhang Y., Lee J., Lee S., Ke Y. (2020). DNA origami guided self-assembly of plasmonic polymers with robust long-range plasmonic resonance. Nano Lett..

[71] Dong J., Zhou Y., Pan J., Zhou C., Wang Q. (2021). Assembling gold nanobipyramids into chiral plasmonic nanostructures with DNA origami. Chem. Commun..

[72] Pan J., Wang X., Zhang J., Zhang Q., Wang Q., Zhou C. (2022). Chirally assembled plasmonic metamolecules from intrinsically chiral nanoparticles. Nano Res..

[73] Martens K., Binkowski F., Nguyen L., Hu L., Govorov A.O., Burger S., Liedl T. (2021). Long- and short-ranged chiral interactions in DNA-assembled plasmonic chains. Nat. Commun..

[74] Zhang W., Ai B., Gu P., Guan Y., Wang Z., Xiao Z., Zhang G. (2021). Plasmonic chiral metamaterials with sub-10 nm nanogaps. ACS Nano.

[75] Yang P., Deng Q., Duan Y., Liu Z., Fang Y., Han L., Che S. (2022). Chiral nanostructured bimetallic au–ag films for enantiomeric discrimination. Adv. Mater. Interfaces.

[76] Kong X.T., Khorashad L.K., Wang Z., Govorov A.O. (2018). Photothermal circular dichroism induced by plasmon resonances in chiral metamaterial absorbers and bolometers. Nano Lett..

[77] Miandashti A.R., Khorashad L.K., Kordesch M.E., Govorov A.O., Richardson H.H. (2020). Experimental and theoretical observation of photothermal chirality in gold nanoparticle helicoids. ACS Nano.

[78] Wen T., Zhang W., Liu S., Hu A., Zhao J., Ye Y., Chen Y., Qiu C.-W., Gong Q., Lu G. (2020). Steering valley-polarized emission of monolayer mos2 sandwiched in plasmonic antennas. Sci. Adv..

[79] Gao H., Chen P.G., Lo T.W., Jin W., Lei D. (2021). Selective excitation of polarization-steered chiral photoluminescence in single plasmonic nanohelicoids. Adv. Funct. Mater..

[80] Liu Z., Ai J., Kumar P., You E., Zhou X., Liu X., Tian Z., Bour P., Duan Y., Han L. (2020). Enantiomeric discrimination by surface-enhanced raman scattering-chiral anisotropy of chiral nanostructured gold films. Angew. Chem. Int. Ed. Engl..

[81] Abdali S., Blanch E.W. (2008). Surface enhanced raman optical activity (seroa). Chem. Soc. Rev..

[82] Ma Y., Cao Z., Hao J., Zhou J., Yang Z., Yang Y., Wei J. (2020). Controlled synthesis of au chiral propellers from seeded growth of au nanoplates for chiral differentiation of biomolecules. J. Phys. Chem. C.

[83] Wen X., Wang S., Liu R., Duan R., Hu S., Jiao T., Zhang L., Liu M. (2022). Selenocystine and photo-irradiation directed growth of helically grooved gold nanoarrows. Small.

[84] Wu F., Tian Y., Luan X., Lv X., Li F., Xu G., Niu W. (2022). Synthesis of chiral au nanocrystals with precise homochiral facets for enantioselective surface chemistry. Nano Lett..

[85] Wang G., Hao C., Ma W., Qu A., Chen C., Xu J., Xu C., Kuang H., Xu L. (2021). Chiral plasmonic triangular nanorings with sers activity for ultrasensitive detection of amyloid proteins in alzheimer’s disease. Adv. Mater..

[86] Yan W., Wang Y., Zhuang H., Zhang J. (2015). DNA-engineered chiroplasmonic heteropyramids for ultrasensitive detection of mercury ion. Biosens. Bioelectron..

[87] Xu Z., Xu L., Liz-Marzán L.M., Ma W., Kotov N.A., Wang L., Kuang H., Xu C. (2013). Sensitive detection of silver ions based on chiroplasmonic assemblies of nanoparticles. Adv. Opt. Mater..

[88] Kuang H., Yin H., Xing C., Xu C. (2013). A sensitive dnazyme-based chiral sensor for lead detection. Materials.

[89] Gao R., Xu L., Hao C., Xu C., Kuang H. (2019). Circular polarized light activated chiral satellite nanoprobes for the imaging and analysis of multiple metal ions in living cells. Angew. Chem. Int. Ed. Engl..

[90] Abbasi S., Khani H. (2017). Highly selective and sensitive method for cu(2+) detection based on chiroptical activity of l-cysteine mediated au nanorod assemblies. Spectrochim. Acta. A. Mol. Biomol. Spectrosc..

[91] Xu L., Gao Y., Kuang H., Liz-Marzan L.M., Xu C. (2018). Microrna-directed intracellular self-assembly of chiral nanorod dimers. Angew. Chem. Int. Ed. Engl..

[92] Ma W., Kuang H., Xu L., Ding L., Xu C., Wang L., Kotov N.A. (2013). Attomolar DNA detection with chiral nanorod assemblies. Nat. Commun..

[93] Yan W., Xu L., Ma W., Liu L., Wang L., Kuang H., Xu C. (2014). Pyramidal sensor platform with reversible chiroptical signals for DNA detection. Small.

[94] Funck T., Nicoli F., Kuzyk A., Liedl T. (2018). Sensing picomolar concentrations of rna using switchable plasmonic chirality. Angew. Chem. Int. Ed. Engl..

[95] Meng D., Ma W., Wu X., Xu C., Kuang H. (2020). DNA-driven two-layer core-satellite gold nanostructures for ultrasensitive microrna detection in living cells. Small.

[96] Zhu F., Li X., Li Y., Yan M., Liu S. (2015). Enantioselective circular dichroism sensing of cysteine and glutathione with gold nanorods. Anal. Chem..

[97] Hao C., Kuang H., Xu L., Liu L., Ma W., Wang L., Xu C. (2013). Chiral supernanostructures for ultrasensitive endonuclease analysis. J. Mater. Chem. B.

[98] Xu L., Xu Z., Ma W., Liu L., Wang L., Kuang H., Xu C. (2013). Highly selective recognition and ultrasensitive quantification of enantiomers. J. Mater. Chem. B.

[99] Zhao H., Bian S., Yang Y., Wu X. (2017). Chiroplasmonic assemblies of gold nanoparticles as a novel method for sensitive detection of alpha-fetoprotein. Microchim. Acta.

[100] Funck T., Liedl T., Bae W. (2019). Dual aptamer-functionalized 3d plasmonic metamolecule for thrombin sensing. Appl. Sci..

[101] Wu X., Xu L., Liu L., Ma W., Yin H., Kuang H., Wang L., Xu C., Kotov N.A. (2013). Unexpected chirality of nanoparticle dimers and ultrasensitive chiroplasmonic bioanalysis. J. Am. Chem. Soc..

[102] Tang L., Li S., Xu L., Ma W., Kuang H., Wang L., Xu C. (2015). Chirality-based au@ag nanorod dimers sensor for ultrasensitive psa detection. ACS Appl. Mater. Interfaces.

[103] Fu P., Sun M., Xu L., Wu X., Liu L., Kuang H., Song S., Xu C. (2016). A self-assembled chiral-aptasensor for atp activity detection. Nanoscale.

[104] Sun M., Xu L., Fu P., Wu X., Kuang H., Liu L., Xu C. (2016). Scissor-like chiral metamolecules for probing intracellular telomerase activity. Adv. Funct. Mater..

[105] Liu F., Li N., Shang Y., Wang Y., Liu Q., Ma Z., Jiang Q., Ding B. (2022). A DNA-based plasmonic nanodevice for cascade signal amplification. Angew. Chem. Int. Ed. Engl..

[106] Huang Y., Nguyen M.K., Natarajan A.K., Nguyen V.H., Kuzyk A. (2018). A DNA origami-based chiral plasmonic sensing device. ACS Appl. Mater. Interfaces.

[107] Sun M., Qu A., Hao C., Wu X., Xu L., Xu C., Kuang H. (2018). Chiral upconversion heterodimers for quantitative analysis and bioimaging of antibiotic-resistant bacteria in vivo. Adv. Mater..

[108] Li S., Xu L., Lu M., Sun M., Xu L., Hao C., Wu X., Xu C., Kuang H. (2021). Metabolic profile of chiral cobalt oxide nanoparticles in vitro and in vivo. Nano Res..

[109] Zhao L., Zhou Y., Niu G., Gao F., Sun Z., Li H., Jiang Y. (2022). Advances in chiral gold nano-assemblies and their bioapplication based on optical properties. Part. Part. Syst. Charact..

[110] Li S., Xu L., Ma W., Wu X., Sun M., Kuang H., Wang L., Kotov N.A., Xu C. (2016). Dual-mode ultrasensitive quantification of microrna in living cells by chiroplasmonic nanopyramids self-assembled from gold and upconversion nanoparticles. J. Am. Chem. Soc..

[111] Ma W., Sun M., Fu P., Li S., Xu L., Kuang H., Xu C. (2017). A chiral-nanoassemblies-enabled strategy for simultaneously profiling surface glycoprotein and microrna in living cells. Adv. Mater..

[112] Sun M., Hao T., Li X., Qu A., Xu L., Hao C., Xu C., Kuang H. (2018). Direct observation of selective autophagy induction in cells and tissues by self-assembled chiral nanodevice. Nat. Commun..

[113] Qu A., Wu X., Li S., Sun M., Xu L., Kuang H., Xu C. (2020). An nir-responsive DNA-mediated nanotetrahedron enhances the clearance of senescent cells. Adv. Mater..

[114] Gao F., Sun M., Ma W., Wu X., Liu L., Kuang H., Xu C. (2017). A singlet oxygen generating agent by chirality-dependent plasmonic shell-satellite nanoassembly. Adv. Mater..

[115] Kumar J., Erana H., Lopez-Martinez E., Claes N., Martin V.F., Solis D.M., Bals S., Cortajarena A.L., Castilla J., Liz-Marzan L.M. (2018). Detection of amyloid fibrils in parkinson’s disease using plasmonic chirality. Proc. Natl. Acad. Sci. USA.

[116] Lu J., Xue Y., Bernardino K., Zhang N.-N., Gomes W.R., Ramesar N.S., Liu S., Hu Z., Sun T., de Moura A.F. (2021). Enhanced optical asymmetry in supramolecular chiroplasmonic assemblies with long-range order. Science.

[117] Sun M., Xu L., Bahng J.H., Kuang H., Alben S., Kotov N.A., Xu C. (2017). Intracellular localization of nanoparticle dimers by chirality reversal. Nat. Commun..

[118] Qu A., Sun M., Kim J.Y., Xu L., Hao C., Ma W., Wu X., Liu X., Kuang H., Kotov N.A. (2021). Stimulation of neural stem cell differentiation by circularly polarized light transduced by chiral nanoassemblies. Nat. Biomed. Eng..

[119] Meng D., Li X., Gao X., Zhang C., Ji Y., Hu Z., Ren L., Wu X. (2021). Constructing chiral gold nanorod oligomers using a spatially separated sergeants-and-soldiers effect. Nanoscale.

[120] Hou S., Zhang H., Yan J., Ji Y., Wen T., Liu W., Hu Z., Wu X. (2015). Plasmonic circular dichroism in side-by-side oligomers of gold nanorods: The influence of chiral molecule location and interparticle distance. Phys. Chem. Chem. Phys..

